# Measurement of treatment burden in patients with multimorbidity in the Netherlands: translation and validation of the Multimorbidity Treatment Burden Questionnaire (NL-MTBQ)

**DOI:** 10.1093/fampra/cmad100

**Published:** 2023-10-25

**Authors:** Loes W S Engels, Tiny van Merode, Monique Heijmans, Juliane Menting, Polly Duncan, Jany Rademakers

**Affiliations:** Department of Family Medicine, School for Public Health and Primary Care (CAPHRI), Maastricht University, Maastricht, The Netherlands; Department of Family Medicine, School for Public Health and Primary Care (CAPHRI), Maastricht University, Maastricht, The Netherlands; Netherlands Institute for Health Services Research (Nivel), Utrecht, The Netherlands; Netherlands Institute for Health Services Research (Nivel), Utrecht, The Netherlands; Centre for Academic Primary Care, University of Bristol, Bristol, United Kingdom; Department of Family Medicine, School for Public Health and Primary Care (CAPHRI), Maastricht University, Maastricht, The Netherlands; Netherlands Institute for Health Services Research (Nivel), Utrecht, The Netherlands

**Keywords:** multimorbidity, patient-reported outcome measure, polypharmacy, psychometrics, quality of life, shared decision-making

## Abstract

**Background:**

Multimorbidity is a growing problem. The number and complexity of (non-)pharmaceutical treatments create a great burden for patients. Treatment burden refers to the perception of the weight of these treatments, and is associated with multimorbidity. Measurement of treatment burden is of great value for optimizing treatment and health-related outcomes.

**Objective:**

We aim to translate and validate the Multimorbidity Treatment Burden Questionnaire (MTBQ) for use in the Dutch population with multimorbidity and explore the level of treatment burden.

**Methods:**

Translating the MTBQ into Dutch included forward–backward translation, piloting, and cognitive interviewing (*n* = 8). Psychometric properties of the questionnaire were assessed in a cross-sectional study of patients with multimorbidity recruited from a panel in the Netherlands (*n* = 959). We examined item properties, dimensionality, internal consistency reliability, and construct validity. The level of treatment burden in the population was assessed.

**Results:**

The mean age among 959 participants with multimorbidity was 69.9 (17–96) years. Median global NL-MTBQ score was 3.85 (interquartile range 0–9.62), representing low treatment burden. Significant floor effects were found for all 13 items of the instrument. Factor analysis supported a single-factor structure. The NL-MTBQ had high internal consistency (*α* = 0.845), and provided good evidence on the construct validity of the scale.

**Conclusion:**

The Dutch version of the 13-item MTBQ is a single-structured, valid, and compact patient-reported outcome measure to assess treatment burden in primary care patients with multimorbidity. It could identify patients experiencing high treatment burden, with great potential to enhance shared decision-making and offer additional support.

**Key messages**

- NL-MTBQ is valid to identify treatment burden in patients with multimorbidity.
- Using the NL-MTBQ in practice gives starting points to start the conversation.
- Future assessment of the instrument and the use of it in practice is suggested.

## Background

In the current time of increasing ageing populations, multimorbidity (the co-occurrence of 2 or more chronic or recurrent health conditions within 1 individual) is a challenge for the health care system.^1–3^ Patients with multimorbidity are at greater risk for safety issues, have poorer quality of life, and the management of multimorbidity is complex and time consuming.^4,5^ The female sex, lower socioeconomic status, and having mental health issues are associated with a higher prevalence of multimorbidity.^3,6^

Present clinical practice guidelines are mostly disease specific which might lead to fragmented care for patients with multimorbidity. Fragmented care carries several risks for treatment, such as inappropriate polypharmacy, poor coordination of care, frequent interaction, and demanding self-management.^4,5,7^ To gain improved insight in the burden entailed by treatment, the concept of treatment burden has been introduced. Treatment burden refers to the perception of the weight of problems and challenges that patients experience, and the self-care practices they must perform to follow the prescribed treatment regimens.^8^ Therefore, treatment burden is an extensive and patient-specific concept involving several domains, such as medication use and impact on daily life.^9^ It is known that patients with multimorbidity often experience higher treatment burden.^4,10,11^

Increased treatment burden is linked to poorer adherence to treatment, which is associated with hospitalization and mortality.^12,13^ Nonetheless, multimorbidity treatment approaches require proper adherence, which means a lifelong burden for patients.^11^ Remarkably, many studies examining treatment burden exclude patients with multiple chronic conditions, while they represent a critical population because of the rising incidence and increased risk of complications and treatment interactions.^10^

Thus, it is important to be able to measure treatment burden in patients with multimorbidity to clarify which aspects of care increase treatment burden and explore how to lower it.^14^ Because of the need to understand the impact of treatment burden and objectify patient experiences, specifically for patients with multimorbidity, Duncan et al. developed and validated a new patient-reported outcome measure, the Multimorbidity Treatment Burden Questionnaire (MTBQ).^14^ Other existing treatment burden assessments have its limitations, they are disease or health care specific, complex, and extensive or only limited too few aspects of treatment burden.^10,14,15^ The MTBQ originally exists of 13 items about treatment burden based on literature review.^14,16–18^ It is a quick and simple tool to screen and identify treatment burden in patients with multimorbidity.^10,19^ Until now, the MTBQ has been successfully translated, psychometrically tested, and validated in a Chinese, Danish, French-Canadian, and German population.^19–22^

The purpose of this study is to translate the MTBQ in Dutch and validate it for use in the Dutch population with multimorbidity. In addition, we aim to explore the level of treatment burden in a group of patients with multimorbidity in the Netherlands using the Dutch version of the MTBQ (NL-MTBQ).

## Methods

The study included 2 steps, conducted from November 2020 to June 2021: (i) translation of the MTBQ into the Dutch language accompanied by piloting and cognitive interviewing, and (ii) assessment of the psychometric properties of the NL-MTBQ and the level of treatment burden in the population.

### Translation and adaptation of the MTBQ

The original MTBQ was translated following the instructions of the World Health Organization.^23^ A bilingual expert group carried out the translation. Two Dutch members of the expert group who master the English language independently performed forward translation of the MTBQ, followed by independent backward translation by a professional translator. The expert group reviewed both backward translations against the original questionnaire, no discrepancies in meaning or any mistranslations were identified. After discussion within the expert group, final consensus was reached on the NL-MTBQ.

The NL-MTBQ was piloted in 8 adult patients with 2 or more chronic diseases recruited from the researcher’s environment and a university hospital. Written informed consent was obtained from all participants. Patient characteristics on sex, age, marital status, number of chronic diseases, type of chronic diseases, number of daily medication, other medication, country of origin, educational level, and informal care were collected to assess the diversity of the study population (Supplementary Table 6). The average time needed to fill in the questionnaire was 2.5 (1–4) min. To assess comprehensibility and cultural adaptation, piloting was accompanied by individual cognitive interviewing (*n* = 8) using a probing interview technique. Patients were interviewed by 1 single researcher to reflect their detailed thoughts about, and problems with the translated questionnaire. Data saturation was reached after interviewing 8 patients. Interviews were audio recorded and manually transcribed ad verbatim. Transcriptions were deductively coded by 2 researchers on the following categories: (mis)understanding, relevance, and suggestion. The coded transcripts were analysed question by question to identify adversities that could potentially lead to misunderstanding of the item. One relevant remark suggested leaving out the abbreviation MTBQ because of ambiguity of its meaning. The expert group agreed on irrelevancy of the abbreviation for the target population. The abbreviation MTBQ was left out.

### Participants and data collection

In this study, cross-sectional data were collected from the National Panel of the Chronically ill and Disabled (NPCD). A nationwide panel, conducted by Nivel, Netherlands Institute for Healthcare Research. The panel consists of roughly 3,500 people who are medically diagnosed with 1, or more chronic somatic diseases (according to the International Classification of Primary Care). These chronic conditions are categorized according to the most common chronic conditions (diabetes, respiratory diseases, cardiovascular diseases, musculoskeletal disorders, cancer, neurological diseases, and digestive diseases). NPCD participants are recruited from a random sample of patients from general practitioners all over the Netherlands. There are no differences in disease characteristics between responders and nonresponders from the random sample. Panel members appear to be representative for the chronically ill population in the Netherlands.^24^ For this study, we selected panel members with multimorbidity (defined as having 2 or more chronic conditions, *n* = 1,251). No absolute rules exist for the sample size needed to validate a questionnaire, a minimum of 5 participants per item is often maintained. However, a larger sample is always better.^25^

Twice a year, NPCD participants receive a questionnaire, either postal or digital, about a variety of aspects of living with a chronic disease and the use of health care. Two reminders were sent. From the NPCD we used data on sex, age, educational level, and multimorbidity. Furthermore, we used data of selected questions about self-rated health and medications from the half-yearly questionnaire for validation (Supplementary Table 7). The NL-MTBQ and the 36-Item Short Form Health Survey (SF-36) were included in the questionnaire in April 2021 for validation purposes.

### Study instruments

#### MTBQ

The MTBQ is a comprehensible questionnaire on the perceived weight of health care tasks and the impact on daily life in patients with multimorbidity. It consists of 10 items and 3 optional items which did not apply in the UK context.^14^ All original 13 items were included for translation (Box 1). Items are scored as followed: 0 (does not apply or not difficult), 1 (a little difficult), 2 (quite difficult), 3 (very difficult), and 4 (extremely difficult). Global MTBQ scores are calculated by multiplying the mean score of all items by 25 to create a score from 0 to 100. These global scores are categorized as determined by Duncan et al.^14^: no treatment burden (0), low treatment burden (<10), medium treatment burden (10–22), and high treatment burden (>22). Psychometric testing of the original MTBQ showed positively skewed scores with floor effects for all items, a single-factor structure explaining 93% of the common variance and high internal consistency (*α* = 0.830).^14^ Due to skewness of global MTBQ scores, it is recommended to report median and interquartile range (IQR) rather than mean and SD.^14^

Box 1. **The original 13 items of the MTBQ.**

Taking lots of medication
Remembering how and when to take medication
Paying for prescriptions, over the counter medication or equipment (optional)
Collecting prescription medication
Monitoring your medical conditions
Arranging appointments with health professionals
Seeing lots of different health professionals
Attending appointments with health professionals
Getting health care in the evenings and at weekends (optional)
Getting help from community services (optional)
 11. Obtaining clear and up-to-date information about your condition
Making recommended lifestyle changes
Having to rely on help from family and friends

#### SF-36

The SF-36 is a validated and commonly used patient-reported survey of self-reported patient health existing of 36 questions with standardized answer options.^26^ The SF-36 is a measure of health status and used as a global measure of health-related quality of life. Total scores were used. The raw scale scores are linearly converted to a 0–100 scale. Higher scores indicate higher levels of well-being/health-related quality of life.^26^ The SF-36 is available in the Dutch language.^27^

### Statistical analysis

Data were analysed using IBM SPSS Statistics for Windows, version 25 (IBM Corp., Armonk, NY). Global scores were calculated as stated above. Descriptive statistics were generated to describe the participants’ characteristics and evaluate the level of treatment burden.

Item properties were analysed by examining the proportion of missing data, the proportion of answers with “does not apply” and the distribution of responses. Floor and ceiling effects were considered present if more than 15% of respondents achieved the lowest (0) or highest (4) score.^28^

To evaluate the dimensionality of the scale an exploratory factor analysis was conducted pairwise using varimax rotation and principal component analysis.^29,30^ The number of extracted factors was determined using a combination of the scree plot, Kaiser’s criterion (eigenvalue greater than 1) and interpretability of domains.^29,31,32^ The eigenvalue of a factor indexes the number of variance accounted for by that factor.^29^ Factor loadings represent the correlation between the items and the common factor. Loading of at least 0.400 was assumed as acceptable.^33^

To test internal consistency reliability, Cronbach’s alpha (0.700–0.950 was assumed acceptable), Crohnbach’s alpha if item deleted and item-total correlations (0.200–0.800 was assumed ideal) were calculated with listwise deletion of missing data.^28,34,35^

Construct validity was examined pairwise by confirming 4 prespecified hypotheses. A confidence interval of 95% was used (*P* > 0.05). We hypothesized a negative association between treatment burden (measured by the global MTBQ score) and health-related quality of life (measured by the SF-36). Next, we expected a positive association between treatment burden and self-reported use of medication, measured by a single question in the Nivel questionnaire. We hypothesized a positive association between treatment burden and the reported number of chronic diseases, as recorded by the Nivel panel. Finally, we assumed a negative association between treatment burden and self-rated health, measured by a single question in the Nivel questionnaire. The associations between treatment burden, measured as global MTBQ score, and these variables were assessed by the Spearman correlation coefficient. A Spearman correlation coefficient greater than 0.500 was considered high, when 0.350–0.500 was considered moderate.^36^

Descriptive statistics were used to calculate both mean and median global NL-MTBQ score and frequencies of treatment burden categories. Differences in global NL-MTBQ scores for age and sex were examined listwise by comparing medians using a nonparametric independent samples test. A confidence interval of 95% was used (*P* > 0.05). For comparing differences in age, due to group size, we converted the existing 4 age groups into 2 age groups.

## Results

### Participant characteristics

A total of 959 (response rate of 77%) respondents suffering from multimorbidity were included in the study. Due to missing values (6.6%) we were able to calculate the global NL-MTBQ score for 896 participants. Participant characteristics are shown in Table 1. The average age was 69.6 years (17–96 years) with slightly more females (51.9%). Less than a third of the participants had a high educational level.

**Table 1.** Characteristics of 959 participants in the validation study.

Characteristics	*N*	%
Sex		
Male	461	48.1
Female	498	51.9
Age (years)		
15–39	17	1.8
40–64	259	27.0
65–74	351	36.6
75 years and older	332	34.6
Educational level^a^		
Low	257	26.8
Medium	391	40.8
High	285	29.7
Missing	26	2.7
Type of chronic diseases		
Cardiovascular disease	267	27.8
Pulmonary disease	178	18.6
Musculoskeletal system disease	109	11.4
Cancer	48	5.0
Diabetes mellitus	126	13.1
Neurological disease	51	5.3
Digestive disease	37	3.9
Other chronic disease	143	14.9
Amount of chronic diseases		
2	510	53.2
3	298	31.1
4 or more	151	15.7
Living status		
Living alone	287	29.9
Living together	660	68.8
Missing	12	1.3

^a^Educational level based on the International Standard Classification of Education (ISCED).^37^

### Statistical analysis

#### Item properties

Proportion of missing data for each question varied between 3.2% and 3.9%. Questions 8, 9, and 10 had a high proportion of “does not apply,” shown in Table 2. High floor effects were found for all items (Table 2).

**Table 2.** Response to the NL-MTBQ (*n* = 959).

Item	Missing *N* (%)	Extremely difficult *N* (%)	Very difficult *N* (%)	Quite difficult *N* (%)	A little difficult *N* (%)	Not difficult *N* (%)	Does not apply *N* (%)
1	31 (3.2)	9 (0.9)	29 (3.0)	64 (6.7)	177 (18.5)	507 (52.9)	142 (14.8)
2	37 (3.9)	1 (0.1)	11 (1.1)	20 (2.1)	78 (8.1)	699 (72.9)	113 (11.8)
3	35 (3.6)	18 (1.9)	24 (2.5)	56 (5.8)	129 (13.5)	473 (49.3)	224 (23.4)
4	34 (3.5)	14 (1.5)	10 (1.0)	13 (1.4)	70 (7.3)	651 (67.9)	167 (17.4)
5	35 (3.6)	7 (0.7)	5 (0.5)	34 (3.5)	132 (13.8)	600 (62.6)	146 (15.2)
6	37 (3.9)	4 (0.4)	6 (0.6)	21 (2.2)	78 (8.1)	703 (73.3)	110 (11.5)
7	34 (3.5)	6 (0.6)	17 (1.8)	35 (3.6)	116 (12.1)	471 (49.1)	280 (29.2)
8	34 (3.5)	2 (0.2)	8 (0.8)	17 (1.8)	60 (6.3)	441 (46.0)	397 (*41.4*)
9	36 (3.8)	6 (0.6)	12 (1.3)	17 (1.8)	55 (5.7)	270 (28.2)	563 (*58.7*)
10	36 (3.8)	1 (0.1)	5 (0.5)	13 (1.4)	40 (4.2)	476 (49.6)	388 (*40.5*)
11	34 (3.5)	4 (0.4)	11 (1.1)	35 (3.6)	98 (10.2)	629 (65.6)	148 (15.4)
12	35 (3.6)	11 (1.1)	41 (4.3)	110 (11.5)	236 (24.6)	358 (37.3)	168 (17.5)
13	31 (3.2)	33 (3.4)	34 (3.5)	82 (8.6)	155 (16.2)	253 (26.4)	371 (38.7)

In italic are percentages ‘does not apply’ of 40% or greater, these can be considered to leave out.

#### Dimensionality

**Fig. 1.**
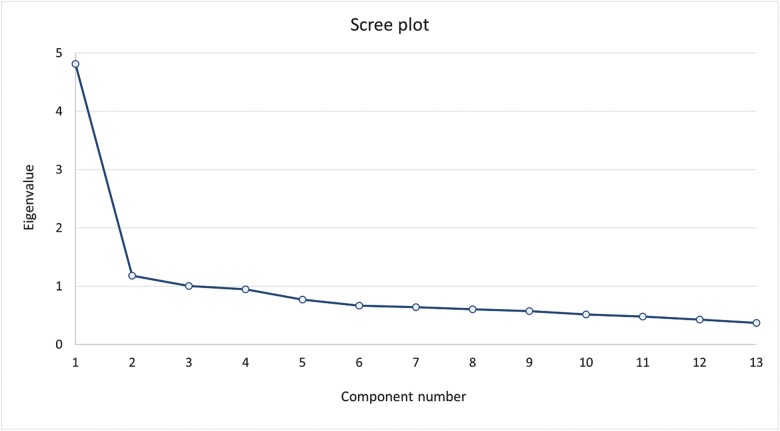
Scree plot of NL-MTBQ.

The Kaiser’s criterion (eigenvalue greater than 1) determined 3 common factors. The eigenvalues of the 3 factors were 4.815, 1.181, and 1.006, respectively. Cross loadings were present for all items (Table 3). However, the scree plot (Fig. 1) showed a sharp drop from factor 1 to 2, suggesting a single-factor solution explaining 37% of total variance. All loadings on factor 1 were consistently greater than 0.400. Items 7, 8, and 10 loaded on factor 2, as well as factor 1 (Table 3). These items all concern interaction with health care providers. Nonetheless, all aspects of treatment burden are connected for this population. One single factor, treatment burden, underlies the items of the NL-MTBQ.

**Table 3.** Factor loadings and eigenvalues for exploratory factory analysis and internal consistency of the NL-MTBQ (*n* = 896).

Item	Factor 1	Factor 2	Factor 3	Cronbach’s alpha if item deleted	Corrected item-total correlation
1	**0.724**	−0.182	−0.312	0.836	0.470
2	**0.688**	−0.141	−0.388	0.837	0.476
3	**0.682**	0.223	−0.127	0.841	0.417
4	**0.653**	0.043	0.252	0.837	0.461
5	**0.642**	−0.136	0.308	0.830	0.575
6	**0.621**	−0.389	−0.334	0.832	0.577
7	**0.577**	**0.452**	−0.188	0.825	0.635
8	**0.569**	**0.508**	0.165	0.834	0.514
9	**0.565**	−0.301	0.203	0.836	0.476
10	**0.551**	**−0.486**	0.328	0.837	0.469
11	**0.550**	0.052	0.340	0.831	0.562
12	**0.550**	0.166	−0.295	0.838	0.466
13	**0.494**	0.296	0.241	0.832	0.572
Eigenvalue	**4.815**	**1.181**	**1.006**		
% of variance explained	37.042	9.085	7.738		
Cumulative % of variance explained	37.042	46.127	**53.865**		

In bold are factor loading values of 0.40 or greater, these are regarded significant.

#### Reliability

The Cronbach’s alpha if item deleted, and corrected item-total correlations are shown in Table 3. All item-total correlations met the recommended minimum of 0.200 and ranged from 0.417 to 0.635. Removing any of the items did not result in significant change of the value of the Cronbach’s alpha. The internal consistency coefficient of the NL-MTBQ was found to be high (*α* = 0.845). This indicates a high level of internal reliability.

#### Construct validity

As predicted, global NL-MTBQ scores were negatively associated with health-related quality of life, measured by the SF-36 (high correlation; *ρ* −0.564, *P* < 0.000, *n* = 765), and self-rated health (moderate correlation; *ρ* −0.440, *P* < 0.000, *n* = 881). Furthermore, global NL-MTBQ scores were positively associated with the amount of different medications participants used (low correlation; *ρ* 0.248, *P* < 0.000, *n* = 868). No association with the reported amount of chronic diseases was found (*ρ* 0.059, *P* = 0.077, *n* = 896). This provides good evidence on the construct validity of the scale.

#### Treatment burden

**Fig. 2.**
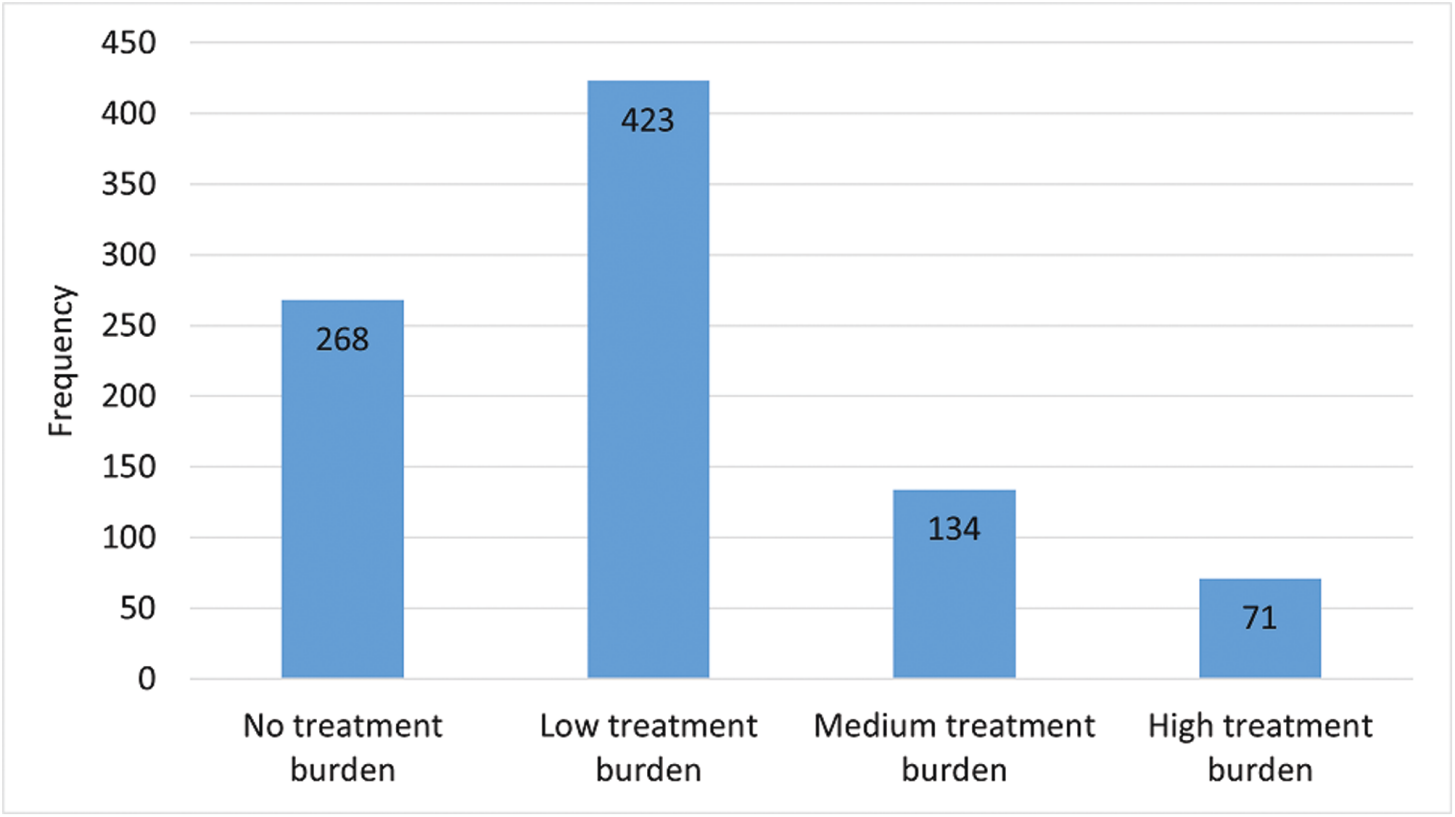
Frequencies of categories of treatment burden in the validation population (*n* = 896).

Mean (SD) global MTBQ score among the validation study population was 7.250 (9.869). The median (IQR) was 3.850 (0–9.62). Both mean and median represent low treatment burden. No significant differences were found between males and females (Table 4). Median global MTBQ scores were significantly higher among younger participants (5.77 IQR 1.92–13.46) when compared with older age (3.85 IQR 0–7.69), shown in Table 4. Most participants demonstrated low treatment burden. Only few participants demonstrated high treatment burden. Frequencies are shown in Fig. 2.

**Table 4.** Median global NL-MTBQ scores compared for age group and sex (*n* = 896).

Age group	Median (IQR)	*N*	*P* value	Sex	Median (IQR)	*N*	*P* value
15–64 years old	5.77 (1.92–13.46)	263	<0.001	Male	3.85 (0–9.62)	432	0.474
65 years and older	3.85 (0–7.69)	633		Female	3.85 (0–9.62)	464	
Total	3.85 (0–9.62)	896		Total	3.85 (0–9.62)	896	

## Discussion

In this study, we have translated and validated the 13-item Dutch Multimorbidity Treatment Burden Questionnaire (NL-MTBQ) for the measurement of treatment burden in the Dutch primary care population with multimorbidity. The NL-MTBQ demonstrated satisfactory psychometric properties with evidence for high internal consistency and good construct validity. The instrument is able to identify patients with high treatment burden.

Three items had high percentages of responses “does not apply” and for future research, these questions could be excluded depending on the target population. Similar to other versions of the MTBQ, high floor effects were found for all items.^14,19–22^ These effects could be explained by homogeneity of the sample frame. The relatively old study population has possibly adapted their daily life in such way that they perceive less burden. Further research is needed to evaluate differences between duration of disease diagnoses. The absence of ceiling effects suggests that the NL-MTBQ may be better for monitoring treatment burden deterioration.

High internal consistency of the NL-MTBQ (*α* = 0.845) was comparable to the original scale (*α* = 0.830),^14^ which indicates a good reliability of the instrument. In accordance with the original scale, we found a single-factor structure (treatment burden).^14^ This supports the usability as an overall measure of treatment burden. Other versions did suggest multiple dimensions, identified as: medication and treatment, medical related, and self-management.^19,21,22^ We do recognize that there are advantages to having subdimensions, which gives the ability to identify specific, more burdensome, aspects.

We found positive associations between treatment burden and the amount of different medications used, lower health-related quality of life and poorer self-rated health. Lack of association between treatment burden and the number of chronic conditions could be explained by the fact that the NPCD used categories (2 chronic conditions, 3, 4, or more) to determine the number of chronic conditions for each panel member. Likewise, data on the number of medications use were only available in categories (no medicines, 1–4, 5, or more). This possibly caused underestimating the associations. For future research, it is recommended to use the exact data instead of categorical data.

On population level we found low treatment burden, comparable to the German version.^22^ Treatment burden was remarkably low when compared with other MTBQ studies.^14,19–21^ These differences could be explained by the variety in organization of health care. Differences in sample composition may also influence treatment burden, the study population was relatively old. Higher perceived treatment burden is associated with being female and being young.^6,14^ Younger people may experience higher treatment burden compared with older people because of the contrast in lifestyle, they more often have to take work, family, or kids into account.

The proportion of missing data was relatively small (6.6%). The absence of data may reduce statistical power, cause bias, or reduce representativeness. Missing data were handled either pairwise or listwise depending on analysis.

### Strengths and limitations

This study has several strengths. First, the translation process was conscientious. In piloting, the NL-MTBQ was found to be compact and user friendly. By using individual cognitive interviewing, we assured that the questionnaire is comprehensible from the patients’ point of view.

Furthermore, the NL-MTBQ has been validated in a large, nationwide, representative sample of adults suffering from multimorbidity. However, information on ethnicity lacked. Participants were recruited from primary care all over the country, which makes it representative for the Dutch primary care population and decreases the risk of selection bias and limitation of generalizability.^38^

However, there are some limitations. Recruiting participants for cognitive interviewing from the researcher’s own environment increases the risk of selection bias. The sample showed little variation in ethnicity and a skew toward well educated. A sample size of 8 interviews is rather small. Nonetheless, data saturation was reached.

Construct validity was tested by determining the association between treatment burden and total SF-36 scores. The SF-36 was not developed as a unidimensional instrument. However, total scores are used in other research despite of the risk for measurement bias.^39^ For future research it is recommended to determine associations with separate dimensions.

Due to the cross-sectional study design, we were not able to test responsiveness. For future research, we suggest analysis of time consistency through test–retest reliability. Other versions of the MTBQ have shown to be time consistent.^19,21^

The original MTBQ scores the response options “not difficult” and “does not apply” as equated, which possibly affects the score distribution. Given the high floor effects, future research may use a revised approach to these considerations.

### Practical implications

Multimorbidity is a complex concept. The 13-item NL-MTBQ is limited to measuring treatment burden and signalling major aspects causing it. In addition, the perception of treatment burden is different for each individual. Administering the NL-MTBQ provides starting points for health care providers to start the conversation, for instance about polypharmacy and propose a comprehensive medication review to lower the burden. Reducing treatment burden is of great importance to improve health-related outcomes.^4,11,40^ Advanced inquiry will be needed to identify specific causes of treatment burden. Further qualitative research could analyse specific domains in more detail. To create a more individual approach for treatment in patients with multimorbidity, evaluation of (individual) treatment burden is of interest for policymakers and could be for instance added in clinical guidelines. This would improve both the aspects of shared decision-making and patient-centred care.^10^ In the future, the NL-MTBQ could possibly contribute to important evidence about sociodemographic factors and health measures related to treatment burden.

## Conclusion

In conclusion, the Dutch version of the Multimorbidity Treatment Burden Questionnaire is a valid, compact patient-reported outcome measure to assess treatment burden in patients suffering from multimorbidity in the Netherlands. It could potentially be used by health care professionals to identify patients that experience a high treatment burden. Furthermore, implementation of the instrument has a great potential to enhance shared decision-making. We do recommend further research to assess the responsiveness of the NL-MTBQ and to evaluate the use of it in practice.

## Supplementary material

Supplementary material is available at *Family Practice* online.

cmad100_suppl_Supplementary_Material

cmad100_suppl_Supplementary_Data

## Data Availability

The data underlying this article will be shared on reasonable request to the corresponding author.
