# Supplementary material for: Measurement of treatment burden in patients with multimorbidity in the Netherlands: translation and validation of the Multimorbidity Treatment Burden Questionnaire (NL-MTBQ)
Source: Fam Pract. 2023 Oct 25;41(6):901–8. doi: 10.1093/fampra/cmad100 (PMC11636562; doi:10.1093/fampra/cmad100)
Supplement: cmad100_suppl_Supplementary_Material [file cmad100_suppl_supplementary_material.pdf]

Supplementary Materials I

Table 6. Sociodemographic sample characteristics of cognitive interviewing (n = 8)

**Participant**	**1**	**2**	**3**	**4**	**5**	**6**	**7**	**8**
Sex	Male	Male	Female	Male	Male	Female	Female	Male
Age	68	67	37	56	65	94	89	97
Marital status	Married	Married	Unmarried	Married	Married	Widow	Widow	Widower
Chronic diseases	4	4	2	3	5	5	3	5
Daily medication	7	5	2	3	6	7	2	7
Origin	NL	NL	NL	NL/IN	NL	NL	NL	NL
*Educational level**	High	High	High	High	Low	Medium	Medium	Low
Informal care	-	-	-	-	Partner	Different providers	-	-

* Educational level based on the International Standard Classification of Education (ISCED)[40].

Supplementary Materials II

Table 7. Selected questions from the NPCD half-yearly questionnaire used for validation of the MTBQ

Original question (and translation)	Original answer options (and translation)
"Hoe veel verschillende soorten medicijnen gebruikt u op dit moment?"<br><br>(How many different medications are you currently taking?)	1 = Geen (None)<br>2 = 1-4 (1-4)<br>3= 5 of meer (5 or more)
"Wat vindt u, over het algemeen genomen, van uw gezondheid?"<br><br>(How do you think, in general, your health status is?)	1 = Uitstekend (Excellent)<br>2 = Zeer goed (Very good)<br>3 = Goed (Good)<br>4 = Matig (Moderate)<br>5 = Slecht (Bad)
